# Leptin regulation of neuronal morphology and hippocampal synaptic function

**DOI:** 10.3389/fnsyn.2013.00003

**Published:** 2013-08-06

**Authors:** Jenni Harvey

**Affiliations:** Division of Neuroscience, Medical Research Institute, Ninewells Hospital and Medical School, University of DundeeDundee, UK

**Keywords:** leptin, synaptic plasticity, morphology, receptor trafficking, hippocampus, AMPA receptor, PTEN

## Abstract

The central actions of the hormone leptin in regulating energy homeostasis via the hypothalamus are well documented. However, evidence is growing that this hormone can also modify the structure and function of synapses throughout the CNS. The hippocampus is a region of the forebrain that plays a crucial role in associative learning and memory and is an area also highly vulnerable to neurodegenerative processes. Recent studies indicate that leptin is a potential cognitive enhancer as it modulates the cellular processes underlying hippocampal-dependent learning and memory including dendritic morphology, glutamate receptor trafficking and activity-dependent synaptic plasticity. Here, we review the recent evidence implicating the hormone leptin as a key regulator of hippocampal synaptic function and discuss the role of leptin receptor-driven lipid signaling pathways involved in this process.

## Introduction

### Leptin receptor signaling

The endocrine hormone, leptin is a 167 amino acid protein that is mainly produced by adipocytes and circulates in the plasma at levels closely correlated with body fat content (Maffei et al., 1995). Although leptin acts on a number of peripheral tissues, leptin also readily targets the CNS, following its transport across the blood brain barrier. Several lines of evidence indicate the expression of leptin mRNA and protein in a number of brain regions, thereby raising the possibility that leptin is also synthesized and released locally in the CNS. The most well documented roles for leptin focus on its actions in the hypothalamus where it participates in the regulation of energy homeostasis, bone formation as well as reproductive function (Spiegelman and Flier, 2001; Karsenty, 2006). However the central actions of leptin are not confined to the hypothalamus. Indeed, numerous studies have detected high levels of leptin receptor expression in extra-hypothalamic brain regions and evidence is growing that leptin has widespread actions throughout the CNS.

The biological actions of leptin are mediated by leptin receptors; members of the class I cytokine receptor superfamily (Tartaglia et al., 1995). The leptin receptor (ObR) is encoded by the diabetes gene (*db*) and alternative splicing of this gene generates six leptin receptor isoforms (ObRa-f) with identical N-terminal domains but vary in the length of their C-terminal region. All the isoforms, except ObRe, have a membrane spanning region consisting of 34 amino acids. ObRe is thought to buffer free circulating leptin levels in the plasma. The membrane spanning ObRs all contain an intracellular proline-rich box which enables association with janus tyrosine kinases, in particular JAK2. However, only the long leptin receptor isoform (ObRb) contains additional intracellular signaling motifs necessary for full JAK-STAT (signal transducer and activation of transcription) signaling. Following leptin binding to ObRb and JAK2 activation, JAK2 associates with and promotes phosphorylation of tyrosine residues within the C-terminal domain. The phosphorylated tyrosine residues enable recruitment and activation of various downstream signaling pathways including the STAT (signal transducers and activators of transcription) family of transcription factors, phosphoinositide 3-kinase as well as adaptor proteins associated with the Ras-Raf-MAPK (mitogen activated protein kinase) pathway. Leptin is capable of activating all of these ObR-driven signaling in central neurons (Harvey, 2003).

### Leptin receptor expression in the CNS

In accordance with its role in regulating energy balance, high levels of leptin receptor expression have been detected on specific hypothalamic nuclei involved in energy homeostasis (Schwartz et al., 1996). Leptin receptors are also expressed throughout the CNS with high levels of expression reported in the amygdala, brainstem and cerebellum. High levels of ObR mRNA and immunoreactivity have also been detected in hippocampal CA1, CA3, and dentate gyrus regions (Mercer et al., 1996; Hâkansson et al., 1998). In hippocampal cultures, ObR expression is localized to principal neurons and glial cells. Moreover, dual labeling studies indicate that ObR is expressed at somato-dendritic regions and also in close proximity to NMDA receptors (Shanley et al., 2002b; O'Malley et al., 2007). Although the main source of leptin is from peripheral tissues, leptin readily gains access to the brain via a saturable transport process at the blood brain barrier (Banks et al., 1996). Short ObR isoforms expressed on brain microvessels are implicated in this process. High levels of ObRa are also expressed at the choroid plexus thereby supporting a possible role for the cerebrospinal fluid in transporting leptin to the CNS. Direct diffusion of leptin to the hypothalamus may also occur as the main leptin-sensitive hypothalamic neurons are located close to the median eminence.

### Leptin regulation of hippocampal synaptic function

The hippocampus is a key brain area involved in learning and memory. Activity-dependent forms of hippocampal synaptic plasticity, such as long-term potentiation (LTP) and long-term depression (LTD) are key cellular events underlying learning, memory and habituation (Bliss and Collingridge, 1993). Recent studies indicate that leptin can modify excitatory synaptic transmission at hippocampal CA1 synapses (Shanley et al., 2001; Xu et al., 2008). Various forms of activity-dependent hippocampal synaptic plasticity are also regulated by this hormone. Indeed, electrophysiological studies have detected impairments in both LTP and LTD at hippocampal CA1 synapses in slices from leptin-insensitive rodents (*fa/fa* rats; *db/db* mice; Li et al., 2002). Defects in hippocampal memory tasks have also been observed in leptin-insensitive rodents (Li et al., 2002; Farr et al., 2006). Administration of leptin directly into the hippocampus not only enhances LTP but also boosts hippocampal-dependent learning and memory (Wayner et al., 2004). Cellular studies performed by Shanley et al. (2001) established that exposure to leptin promotes the conversion of short-term potentiation (STP) into LTP in acute hippocampal slices.

**Figure 1:**
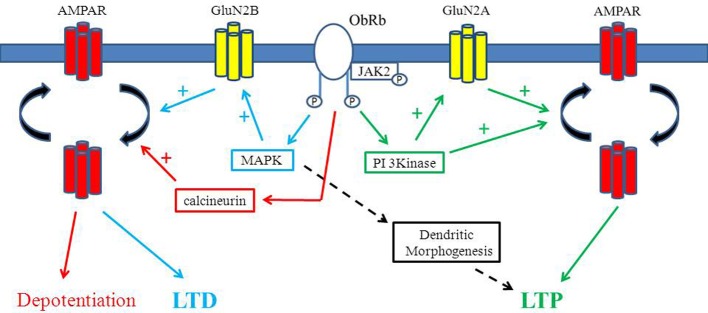
**Divergent effects of leptin on hippocampal synaptic function.** Schematic representation of the key signaling pathways that contributes to the diverse effects of leptin in the hippocampus. Activation of leptin receptors triggers PI 3-kinase stimulation which results in AMPA receptor exocytosis and a sustained increased in synaptic efficacy (leptin-induced LTP). Leptin driven stimulation of PI 3-kinase also enhances GluN2A activity which in turn promotes AMPA receptor delivery to synapses and subsequent LTP at adult hippocampal CA1 synapses. In contrast, at early stages of postnatal development, leptin dependent activation of the ERK (MAPK) signaling cascade facilitates GluN2B-mediated responses resulting in either persistent (LTD) or transient depressions in excitatory synaptic transmission. Leptin is also capable of depotentiating hippocampal synapses via a process involving the activation of calcineurin and subsequent endocytosis of GluA2-lacking AMPA receptors. Rapid alterations in dendritic morphology are also evoked by leptin that are mediated by the MAPK (ERK) signaling cascade.

Although NMDA receptors contribute little to basal synaptic transmission, activation of NMDA receptors is essential for the induction of hippocampal LTP (Collingridge et al., 1983). Like other hormones and growth factors, leptin potently enhances NMDA receptor function which contributes to its ability to facilitate LTP. Indeed, leptin is capable of enhancing Ca^2+^ influx via native NMDA receptor channels in hippocampal cultures and NMDA evoked currents in *Xenopus* oocytes expressing recombinant NMDA receptors (Shanley et al., 2001; Harvey et al., 2005). Conversely, other studies have reported either no effect or attenuation of NMDA responses by leptin in hippocampal neurons (Oomura et al., 2006) which may be due to differences in the experimental approach or age of tissue used. Nevertheless, more recent studies support a crucial role for NMDA receptors, as distinct GluN2 subunits play a key role in leptin's effects on excitatory synaptic transmission at different stages of postnatal development and aging (Moult and Harvey, 2011). In addition, divergent signaling pathways are implicated in the age-dependent effects of leptin such that the ERK pathway mediates the synaptic depressions evoked by leptin early in postnatal development whereas leptin-driven increases in excitatory synaptic strength in adult are PI 3-kinase dependent (Moult and Harvey, 2011). Previous studies revealed that distinct signaling pathways link leptin receptors to facilitation of molecularly distinct NMDA receptors in cerebellar granule cells (Irving et al., 2006). Thus, early in postnatal development it is possible that leptin driven ERK activation facilitates GluN2B-mediated responses thereby resulting in either persistent or transient depressions in excitatory synaptic transmission. Conversely in adult hippocampus, leptin increases GluN2A-mediated responses via PI 3-kinase which in turn promotes a long lasting increase in synaptic efficacy (Moult and Harvey, 2011; Figure 1).

### Leptin-induced LTD

Two main forms of LTD occur at mammalian central synapses that are generated by the synaptic activation of NMDA and metabotropic glutamate receptors, respectively (Massey and Bashir, 2007). Several studies have shown that hormones and growth factors are also capable of inducing novel forms of LTD. Similarly, under conditions of enhanced excitability, exposure to leptin results in the induction of a novel form of NMDA receptor-dependent hippocampal LTD (Durakoglugil et al., 2005). Leptin-induced LTD shares similar expression mechanisms to activity-dependent LTD as LTD induced by low frequency stimulation (LFS) occludes leptin-induced LTD (Durakoglugil et al., 2005). Moreover, like *de novo* LTD, leptin-induced LTD has a postsynaptic locus of expression as no alteration in the paired pulse ratio was observed during leptin-induced LTD. Moreover, inhibition of PI 3-kinase increased the magnitude of leptin-induced LTD suggesting that PI 3-kinase negatively regulates this process. Similarly, inhibitors of serine/threonine protein phosphatase 1/2A, but not 2B, also enhanced the synaptic depression induced by leptin, indicating that leptin-induced LTD is negatively regulated by phosphatase 1/2A (Durakoglugil et al., 2005).

### Leptin reverses established LTP

It is known that LTP can be readily reversed shortly after induction via a process known as depotentiation. This phenomenon is thought to boost the capacity of neuronal networks to store information by preventing saturation of potentiated synapses. Several studies indicate that application of theta burst stimuli or LFS can reverse LTP at hippocampal CA1 synapses (Bashir and Collingridge, 1994). Recent evidence indicates that leptin can also reverse established LTP, in a time and concentration-dependent manner (Moult et al., 2009). The ability of leptin to depotentiate hippocampal CA1 synapses is not associated with any change in paired-pulse ratio or coefficient of variance (CV), suggesting a postsynaptic expression mechanism. Leptin-induced depotentiation also requires the activation of NMDA receptors as blockade of NMDA receptors with D-AP5 prevented the effects of leptin. A decrease in the rectification properties of synaptic AMPA receptors accompanied leptin- induced depotentiation. Moreover the effects of leptin were mirrored by application of philanthotoxin, a selective inhibitor of GluA2-lacking AMPA receptors, indicating that a reduction in the density of GluA2-lacking AMPA receptors at hippocampal synapses underlies leptin-induced depotentiation (Moult et al., 2009). The involvement of AMPA receptor internalization in leptin-induced depotentiation displays parallels to other studies as removal of AMPA receptors from synapses also contributes to the reversal of hippocampal LTP by LFS, mGluRs, and neuregulin (Zho et al., 2002; Kwon et al., 2005).

Previous studies have identified a role for the stress-activated protein kinase JNK (c-Jun N-terminal kinase) in LFS-induced depotentiation (Zhu et al., 2005). In contrast, however, treatment of slices with selective inhibitors of JNK failed to alter the magnitude of leptin-induced depotentiation suggesting involvement of a JNK-independent process (Moult et al., 2009). However, calcineurin (protein phosphatase 2B) is implicated in NMDA receptor-driven removal of GluA2-lacking AMPA receptors from synapses (Beattie et al., 2000). In accordance with this, leptin-induced depotentiation is blocked by selective inhibitors of calcineurin, indicating a role for PP2B in this process (Figure 1). As dephosphorylation of GluA1 on Ser845 is pivotal for NMDA receptor-driven AMPA receptor endocytosis (Man et al., 2007), it is likely that leptin promotes the activation of calcineurin and subsequent dephosphorylation of GluR1 which in turn results in removal of GluA2-lacking AMPA receptors from hippocampal synapses.

### Leptin evokes a novel form of LTP in adult hippocampus

Application of leptin to acute hippocampal slices from juvenile (2–3 weeks old) rats results in a transient and readily reversible depression of excitatory synaptic transmission (Shanley et al., 2001; Xu et al., 2008). Conversely, in adult leptin evokes a long lasting increase in hippocampal excitatory synaptic strength (leptin-induced LTP) which is sustained following washout of leptin (Moult et al., 2010). Activation of leptin receptors is crucial for leptin-induced LTP as leptin has no effect in slices from Zucker *fa/fa* rats, whereas robust LTP is observed in Zucker lean animals. NMDA receptor activation is also essential as NMDA receptor blockade with D-AP5 prevents leptin-induced LTP (Moult et al., 2010). The ability of leptin to induce LTP in adult is likely to be mediated by GluN2A containing NMDA receptors as the leptin-driven increase in synaptic efficacy is completely blocked by NVP-AAM077, an NMDA receptor antagonist with preferential selectivity for GluN2A subunits (Auberson et al., 2002), but is unaffected by inhibition of GluN2B subunits with ifenprodil (Moult and Harvey, 2011; Figure 1).

Activation of NMDA receptors is pivotal for the induction of hippocampal LTP (Collingridge et al., 1983) as well as promoting AMPA receptor trafficking to synapses during LTP (Collingridge et al., 2004). Recent evidence indicates that hippocampal synaptic plasticity is also associated with alterations in the subunit content of AMPA receptors as synaptic activity increases the density of synaptic GluA2-lacking AMPA receptors (Isaac et al., 2007). Alterations in the molecular composition of AMPA receptors also accompany leptin-induced LTP as AMPA receptor rectification increases after exposure to leptin whereas application of philanthotoxin to block GluA2-lacking AMPA receptors reverses the leptin-induced increase in synaptic efficacy (Moult et al., 2010). The surface expression of GluA1, but not GluA2, subunits is also elevated in hippocampal slices and cultured neurons treated with low nanomolar concentrations of leptin.

PI 3-kinase, which catalyses phosphorylation of PtdIns(45)P_2_ into PtdIns(3,4,5)P_3_, is not only an integral part of neuronal leptin receptor-driven signaling but it is also critical for trafficking AMPA receptors to hippocampal synapses during LTP (Man et al., 2003). Similarly, the ability of leptin to increase GluA1 surface expression involves activation of PI 3-kinase as an increase in PtdIns(3,4,5)P_3_ levels accompanied the effects of leptin and the leptin-driven increase in both GluA1 expression and PtdIns(3,4,5)P_3_ levels were blocked by selective PI 3-kinase inhibitors. However, PtdIns(3,4,5)P_3_ levels are also controlled by PTEN, a phosphatase that promotes conversion of PtdIns(3,4,5)P_3_ to PtdIns(4,5)P_2_, and thus antagonizes PI 3-kinase activity. In support of a role for PTEN, the leptin-driven increase in GluA1 surface expression is combined with an elevation in P366-PTEN staining in hippocampal cultures. Moreover, application of leptin to hippocampal slices resulted in a robust increase in the phosphorylation of PTEN (Moult et al., 2010). In accordance with studies revealing that casein kinase 2 (CK2) phosphorylates PTEN, inhibition of CK2 prevented the effects of leptin on GluA1 surface expression, PTEN phosphorylation and excitatory synaptic transmission. Thus, it is feasible that leptin activates CK2 which phosphorylates and inhibits PTEN thereby increasing PtdIns(3,4,5)P_3_ levels and in turn driving alterations in AMPA receptor trafficking and excitatory synaptic strength.

In further support of a role for PTEN, expression of dominant-negative PTEN mutants (C124S or G129E) in neurons or pharmacological inhibition of PTEN with the phosphatase inhibitor bisperoxovanadium (Bpv) mirror and occlude the effects of leptin on GluA1 trafficking and hippocampal synaptic function (Moult et al., 2010). As inhibition of PTEN leads to an elevation in PtdIns(3,4,5)P_3_ levels, it is likely that alterations in the levels of this inositol lipid are crucial for modifying AMPA receptor trafficking processes. Recent evidence supports this possibility as the availability of PtdIns(3,4,5)P_3_ is reported to maintain AMPA receptor clustering and synaptic function at hippocampal synapses (Arendt et al., 2010). Although the exact role of PtdIns(3,4,5)P_3_ remains to be determined, it is likely that PtdIns(3,4,5)P_3_ influences AMPA receptor trafficking by either altering actin cytoskeletal dynamics (Zhou et al., 2001) or promoting Akt-driven inhibition of glycogen synthase kinase 3 (GSK-3; Peineau et al., 2007).

### Leptin promotes morphological changes in hippocampal dendrites

Alterations in the morphology of dendrites and spines are reported to occur after hippocampal activity-dependent synaptic plasticity and these structural changes are thought to contribute to modifications in excitatory synaptic strength (Maletic-Savatic et al., 1999). In addition, several hormones are reported to induce rapid morphological changes in neurons which can enable further regulation of neuronal connectivity and synaptic strength. Likewise, exposure of hippocampal neurons to leptin results in marked changes in the density and motility of dendritic filopodia within a few minutes (O'Malley et al., 2007). Time lapse confocal microscopy studies also reveal that leptin rapidly increases the density and motility of dendritic filopodia in hippocampal neurons transfected with a cytosolic EGFP construct (O'Malley et al., 2007). The actin cytoskeleton is reported to play a key role in the morphological changes that occur during synaptic plasticity (Matus, 2000; Smart and Halpain, 2000). Thus, as leptin can rapidly alter the structure of actin filaments in hippocampal neurons and hypothalamic cell lines (O'Malley et al., 2005; Ning et al., 2006) it is feasible that leptin-driven formation of new filopodia involves alterations in actin cytoskeletal dynamics. Indeed, the leptin-dependent increase in dendritic filopodia is accompanied by a significant reduction in polymerized actin staining in proximal dendrites, which is consistent with leptin driven redistribution of actin filaments from the dendritic shaft to dendritic filopodia.

It has been shown that activation of synaptic NMDA receptors stimulates the appearance of dendritic protrusions (Maletic-Savatic et al., 1999) and neuronal exposure to glutamate regulates the formation and motility of dendritic filopodia (McKinney et al., 1999; Fischer et al., 2000). In accordance with these studies, the synaptic activation of NMDA receptors is pivotal for the effects of leptin as the morphology of dendrites is unaffected by leptin in neurons treated with either tetrodotoxin or the competitive NMDA receptor antagonist, D-AP5. However, blockade of GluN2B containing NMDA receptors with ifenprodil failed to alter the leptin-driven structural changes indicating the likely involvement of GluN2A containing NMDA receptors (O'Malley et al., 2007). As GluN2A subunits are predominantly expressed at synaptic loci, it is likely that the activation of synaptic, as oppose to extrasynaptic NMDA receptors is pivotal for the morphological changes induced by leptin. It is known that PI 3-kinase and MAPK are two of the major signaling pathways activated by neuronal leptin receptors (Niswender et al., 2001; Harvey, 2003; Irving et al., 2006). Moreover, the ability of leptin to enhance NMDA receptor function in the hippocampus is mediated by both PI 3-kinase and MAPK (Shanley et al., 2001). However, only the MAPK signaling cascade is required for the rapid structural changes induced by leptin as inhibitors of MEK but not PI 3-kinase attenuated the effects of leptin on dendritic morphology (Figure 1). Our previous studies indicate that actin filament reorganization underlies the activation of large-conductance Ca^2+^-activated K^+^ (BK) channels by leptin (Shanley et al., 2002a; O'Malley et al., 2005). However, a PI 3-kinase, rather than MAPK- dependent process mediates actin filament disruption and subsequent activation of BK channels by leptin (Shanley et al., 2002a). Activation of PI 3-kinase by leptin has been shown to trigger localized elevations in phosphatidylinositol-3,4,5-triphosphate [PtdIns(3,4,5)P_3_; (O'Malley et al., 2005)] levels in close proximity to the plasma membrane. Thus, it is likely that leptin-driven reorganization of actin filaments via this pathway occurs in a highly compartmentalized manner. In contrast, however, activation of MAPK by leptin may promote more extensive alterations in the actin cytoskeleton as numerous downstream targets for MAPK are widely expressed in dendritic and somatic regions in hippocampal neurons (Thomas and Huganir, 2004).

During the earliest stages of synaptogenesis there is increased movement and extension of dendritic filopodia (Fiala et al., 1998; Munno and Syed, 2003). Dendritic filopodia are also thought to play an active role in initiating synaptic contacts during synaptogenesis (Ziv and Smith, 1996). Thus, it is feasible that leptin, by increasing the density and motility of dendritic filopodia, alters the number of synaptic connections. Indeed, O'Malley et al. (2007) evaluated if leptin altered synaptic connectivity using immunocytochemical approaches to assay the relative number of presynaptic terminals. Exposure of hippocampal cultures to leptin for 30 min resulted in elevations in the density of actin-rich spines and also synapsin-1-positive puncta, which is consistent with leptin increasing the number of hippocampal synapses (O'Malley et al., 2007).

The structural changes induced by leptin display many parallels to the morphological changes observed following activity-dependent synaptic plasticity. Indeed, increases in the density of actin-rich spines and dendritic filopodia occur following hippocampal LTP, which parallels the morphological changes induced by leptin in hippocampal neurons (Maletic-Savatic et al., 1999; Fukazawa et al., 2003). However, the appearance of dendritic filopodia has also been linked to the induction of hippocampal LTD (Bourne and Harris, 2007). Thus, the possibility that leptin-driven alterations in dendritic filopodia play a role in the persistent synaptic depressions induced by leptin cannot be excluded. The time course of the morphological changes induced by leptin is similar to those associated with LTP (Maletic-Savatic et al., 1999), and like LTP, the rapid growth of dendritic filopodia induced by leptin requires the synaptic activation of NMDA receptors (Maletic-Savatic et al., 1999). Activation of MAPK is implicated in the structural changes associated with activity-dependent synaptic plasticity as NMDA receptor activation promotes increases in MAPK activity and dendritic spine density in hippocampal neurons (Goldin and Segal, 2003), whereas new hippocampal dendritic spines and filopodia are formed after sustained activation of MAPK (Wu et al., 2001). Similarly, a role for MAPK is also thought to underlie the effects of leptin as leptin failed to induce structural changes following blockade of MAPK activation (O'Malley et al., 2007).

In addition to regulating hippocampal neuron morphology, recent evidence indicates that leptin has the capacity to alter the morphology of neuronal populations. Thus, leptin promotes neurite outgrowth from hypothalamic (Bouret et al., 2004) and cerebellar purkinje neurons (Oldreive et al., 2008). Exposure to leptin also alters the size of axonal growth cones in cortical neurons (Valerio et al., 2006). Studies in leptin deficient (*ob/ob*) mice have identified significant alterations in the density of both excitatory and inhibitory synapses compared to wild type mice (Pinto et al., 2004). Furthermore, these alterations are normalized within 6 h of systemic administration of leptin to *ob/ob* mice. However, the morphological changes induced by leptin in hypothalamic, cerebellar, and cortical neurons occur on a much slower time scale than in the hippocampus, as structural changes were only evident after treatment with leptin for several hours (Bouret et al., 2004; Valerio et al., 2006; Oldreive et al., 2008).

### Potential cross-talk between leptin and other hormonal systems in the hippocampus

Numerous studies indicate that leptin is an important modulator of activity-dependent synaptic plasticity at different stages of postnatal development and aging. However, although numerous growth factors and hormones are capable of regulating hippocampal synaptic function, it remains to be determined if the effects of leptin are influenced by other hormonal systems. It is feasible that there is not only convergence of function but also potential cross-talk between hormonal systems at the level of the signal transduction pathways. Indeed, the endocrine hormone insulin that is secreted by pancreatic beta cells in response to elevated glucose levels displays many parallels to leptin in terms of its central actions and also the signaling pathways that it activates. Thus, in a manner similar to leptin, insulin reduces food intake when administered centrally (Schwartz et al., 1992). Insulin derived from peripheral sources is also readily transported into the brain where it can regulate hippocampal synaptic function. Indeed, at hippocampal CA1 synapses, insulin promotes endocytosis of GluA2 and subsequent induction of a novel form of NMDA receptor-dependent LTD (Man et al., 2000; Ahmadian et al., 2004; Huang et al., 2004). Like leptin, insulin is capable of facilitating NMDA responses and increasing NMDA receptor exocytosis (Liu et al., 1995; Skeberdis et al., 2001). Furthermore, exposure of hippocampal neurons to insulin stimulates an increase in the cell surface expression of GluA1 (Man et al., 2000; Passafaro et al., 2001), which parallels the actions of leptin on GluA1 trafficking processes (Moult et al., 2010). Thus, given the overlapping functional effects of leptin and insulin in the hippocampus, it is likely that there is also cross-talk between the hormonal driven signaling pathways in this brain region. This possibility is supported by studies in hypothalamic neurons as insulin not only mimics the ability of leptin to activate ATP-sensitive K^+^ (K_*ATP*_) channels but channel activation by both hormones involves a PI 3-kinase-dependent process (Harvey et al., 2000; Spanswick et al., 2000). PI3-Kinase is also implicated in the activation of hypothalamic large-conductance Ca^2+^-activated K^+^ (BK) channels by leptin and insulin (Yang et al., 2010). Thus, modulation of PI3K signaling may act as a point of convergence for the regulation of hippocampal synaptic function by the leptin and insulin hormonal systems.

Both the PI 3-Kinase and the Ras/Raf/MEK signaling pathways are activated by other class I cytokines, including interleukin 6 (IL-6). Moreover, as evidence is growing that IL-6 is a potent regulator of hippocampal synaptic function (Tancredi et al., 2000; Nelson et al., 2012), the possibility that there is also convergence between leptin and IL-6 driven signaling cascades in hippocampal neurons cannot be excluded. It is also feasible that other hormonal systems indirectly influence leptin-driven regulation of hippocampal synaptic function by modulating leptin levels. In support of this possibility, insulin-like growth factor-1 (IGF-1) is reported to increase the expression of leptin in organotypic hippocampal slices (Marwarha et al., 2011). Although there is some evidence supporting potential interactions between leptin and other hormonal systems in the hippocampus, it is clear that a greater understanding of the interplay between different hormones is required. In particular it is key that the complex cellular events underlying synaptic plasticity and how such processes are modulated by hormonal systems are more fully understood.

## Conclusion

It is well established that the endocrine hormone leptin regulates many central processes including energy homeostasis. However, evidence is growing that the structure and function of hippocampal CA1 synapses is also markedly influenced by leptin. Recent studies indicate that leptin has cognitive enhancing properties as it rapidly alters glutamate receptor trafficking, dendritic morphology and different forms of activity-dependent hippocampal synaptic plasticity. Regulation of NMDA receptor activity by leptin appears to be key for its ability to influence multiple aspects of hippocampal synaptic function, although it is not entirely clear how leptin-driven activation of NMDARs leads to such opposing effects on hippocampal synaptic function. However, emerging studies indicate not only that distinct NMDA receptor subunits are pivotal for leptin's effects on excitatory synaptic transmission, but also that distinct signaling pathways couple leptin receptors to molecularly distinct NMDA receptors at different developmental stages. Although it is established that leptin plays a pivotal role in normal brain function, disruption of the leptin system is also linked to neurodegenerative disorders, like Alzheimer's disease. Thus, the ability of leptin to regulate neuronal morphology and synaptic efficacy is likely to have important implications not only in health but also in diseases associated with leptin dysfunction.

### Conflict of interest statement

The author declares that the research was conducted in the absence of any commercial or financial relationships that could be construed as a potential conflict of interest.
